# Cerebrospinal fluid total tau concentration predicts clinical phenotype in Huntington's disease

**DOI:** 10.1111/jnc.13719

**Published:** 2016-09-20

**Authors:** Filipe Brogueira Rodrigues, Lauren Byrne, Peter McColgan, Nicola Robertson, Sarah J. Tabrizi, Blair R. Leavitt, Henrik Zetterberg, Edward J. Wild

**Affiliations:** ^1^ Huntington's Disease CentreInstitute of NeurologyUniversity College LondonLondonUK; ^2^ Centre for Molecular Medicine and TherapeuticsDepartment of Medical GeneticsUniversity of British ColumbiaVancouverBCCanada; ^3^ Institute of Neuroscience and PhysiologyDepartment of Psychiatry and Neurochemistrythe Sahlgrenska Academy at the University of GothenburgMölndalSweden; ^4^ Department of Molecular NeuroscienceInstitute of NeurologyUniversity College LondonLondonUK

**Keywords:** biomarkers, cerebrospinal fluid, Huntington disease, pilot projects, tau proteins

## Abstract

Huntington's disease (HD) is a hereditary neurodegenerative condition with no therapeutic intervention known to alter disease progression, but several trials are ongoing and biomarkers of disease progression are needed. Tau is an axonal protein, often altered in neurodegeneration, and recent studies pointed out its role on HD neuropathology. Our goal was to study whether cerebrospinal fluid (CSF) tau is a biomarker of disease progression in HD. After informed consent, healthy controls, pre‐symptomatic and symptomatic gene expansion carriers were recruited from two HD clinics. All participants underwent assessment with the Unified HD Rating Scale ’99 (UHDRS). CSF was obtained according to a standardized lumbar puncture protocol. CSF tau was quantified using enzyme‐linked immunosorbent assay. Comparisons between two groups were tested using ancova. Pearson's correlation coefficients were calculated for disease progression. Significance level was defined as *p* < 0.05. Seventy‐six participants were included in this cross‐sectional multicenter international pilot study. Age‐adjusted CSF tau was significantly elevated in gene expansion carriers compared with healthy controls (*p* = 0.002). UHDRS total functional capacity was significantly correlated with CSF tau (*r* = −0.29, *p* = 0.004) after adjustment for age, and UHDRS total motor score was significantly correlated with CSF tau after adjustment for age (*r* = 0.32, *p* = 0.002). Several UHDRS cognitive tasks were also significantly correlated with CST total tau after age‐adjustment. This study confirms that CSF tau concentrations in HD gene mutation carriers are increased compared with healthy controls and reports for the first time that CSF tau concentration is associated with phenotypic variability in HD. These conclusions strengthen the case for CSF tau as a biomarker in HD.

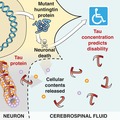

In the era of novel targeted approaches to Huntington's disease, reliable biomarkers are needed. We quantified Tau protein, a marker of neuronal death, in cerebrospinal fluid and found it was increased in patients with Huntington's disease and predicted motor, cognitive, and functional disability in patients. It is therefore likely to be a biomarker of disease progression, and possibly of therapeutic response.

**Read the Editorial Highlight** for this article on page 9.

**Abbreviations used**
α: alfa
β: beta
ANCOVA: analysis of covariance
CAG: cytosine‐adenine‐guanine
CSF: cerebrospinal fluid
DCL: diagnostic confidence level
HD: Huntington's disease
p: p‐value
r: Pearson's coefficient of correlation
SD: standard deviation
TFC: UHDRS total functional capacity
TMS: UHDRS total motor score
UCL: University College London
UHDRS: Unified HD Rating Scale ’99
UK: United Kingdom

Huntington's disease (HD) is a hereditary autosomal dominant neurodegenerative condition that affects movement control, behavior, and cognition (Bates *et al*. 2015). No therapeutic intervention has been found to alter disease progression but several drugs are currently being developed and tested (Bates *et al*. 2015). The progression of HD is slow, with half of the patients surviving at least 24 years after the motor diagnosis (Rodrigues *et al*. 2014). This makes studying the efficacy of disease‐modifying compounds challenging.

The development of sensitive biomarkers of disease progression that reflect neuropathology and treatment response is of vital importance to empower clinical trials.

Tau is a protein with microtubule‐stabilizing functions, which is mainly confined to axons, and when abnormally assembled or aggregated, is associated with multiple neurodegenerative conditions, Alzheimer's disease in particular (Spillantini and Goedert 2013). Indeed, it has been shown that tau concentration in cerebrospinal fluid (CSF) of patients with Alzheimer's disease is associated with disease progression (Blom *et al*. 2009). It has been shown that HD patients have higher CSF total tau concentrations compared with healthy controls (Constantinescu *et al*. 2011), but no associations with phenotype or independent validation were made.

Recently, two independent groups showed that human HD, while principally a ‘huntingtinopathy’, was also in effect a secondary tauopathy, with tau isoform imbalances, increased total tau, nuclear tau deposits, and tau co‐localization with mHTT (Fernandez‐Nogales *et al*. 2014; Vuono *et al*. 2015). Given all this, we set out to study whether CSF total tau is a biomarker of disease progression.

## Methods

### Ethical approval

All human experiments were performed in accordance with the declaration of Helsinki and approved by the University College London (UCL)/UCL Hospitals Joint Research Ethics Committee (UK participants) and the University of British Columbia Clinical Research Ethics Board, as appropriate. All subjects gave informed written consent.

### Participants

Subjects were recruited from two HD multidisciplinary clinics, one in London, United Kingdom and other in Vancouver, Canada. Healthy controls, pre‐symptomatic gene expansion carriers, and symptomatic gene expansion carriers were included. Individual with infectious, inflammatory, or other concomitant CNS disorders or significant comorbidities were excluded. Healthy controls were defined as individuals without family history of HD and without symptoms compatible with HD, or without a cytosine‐adenine‐guanine (CAG)‐expanded allele of the HD gene. Asymptomatic gene expansion carriers were defined as individuals with a CAG‐expanded allele of the HD gene and with a diagnostic confidence level inferior to 4. Symptomatic gene expansion carriers were defined as individuals with a CAG‐expanded allele of the HD gene and with a diagnostic confidence level of 4 (Reilmann *et al*. 2014).

### Clinical assessment

All participants underwent a collection of demographic and clinical data as well as Unified HD Rating Scale ’99 (UHDRS) (Huntington's disease study group 1996), assessed by an experienced neurologist. Age, gender, CAG repeat length of gene expansion carriers, disease stage (Bates *et al*. 2014), total functional capacity (Shoulson and Fahn 1979) (TFC), and total motor score (Huntington's disease study group 1996) (TMS) were recorded. The UHDRS cognitive tasks (Symbol‐digit modality test, Stroop color matching task, Stroop word matching task, and Stroop interference task) were also recorded in the Vancouver cohort (Huntington's disease study group 1996). Disease burden was calculated according to CAG repeat number and age (Penney *et al*. 1997). Patients with motor abnormalities were defined as having early, moderate, or advanced disease using the TFC scale (13–7, early; 6–4, moderate; 3–0, advanced) assessed by experienced clinical raters (Bates *et al*. 2014).

### CSF sample collection and storage

CSF was obtained by lumbar puncture performed between 9 : 00 and 11 : 00 am after fasting from midnight (water was permitted), examined by microscopy, and centrifuged to remove cells, and the acellular portion was frozen at −80°C. Further details were as previously published (Wild *et al*. 2015). Hemoglobin concentration using multiwavelength spectrophotometric readings was assessed to determine CSF contamination by blood.

### CSF total tau quantification

CSF total tau was quantified using the INNOTEST enzyme‐linked immunosorbent assay according to the manufacturer's instructions (Fujirebio, Ghent, Belgium) in one round of experiments using one batch of reagents by board‐certified laboratory technicians who were blinded to clinical data.

### Statistical analysis

Statistical analysis was performed with Stata version 14 software (StataCorp, College Station, Texas, USA). Potentially confounding demographic variables (age and gender) were examined in preliminary analyses. Total tau distribution was tested for normality using skewness and kurtosis, Shapiro–Wilk and Shapiro–Francia tests. Comparisons between two groups adjusted for covariates were tested using ancova. To study the association of total tau with disease progression, we calculated Pearson's and partial correlations coefficients. Bootstrapping with 1000 repetitions was applied to non‐normal variables. Significance level was defined as *p* < 0.05.

## Results

**Figure 1:**
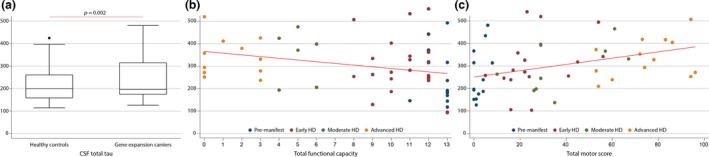
(a) Comparison between healthy controls versus gene expansion carriers; (b) Association between total tau and total functional capacity; (c) Association between total tau and Unified Huntington's Disease Rating Scale total motor score.

We conducted a cross‐sectional multicenter international pilot study of 76 participants (mean age 47.9; standard deviation [SD] 13.1; range 23–72 years; Table 1). Of these, 24 (31.6%) were healthy controls and 52 (68.4%) were gene expansion carriers – 13 (17.1%) pre‐manifest, 22 (29.0%) early HD, 6 (7.9%) moderate HD, and 11 (14.5%) advanced HD. The gene expansion group had a mean CAG repeat length of 43.8 (SD 3.9, range 36–63), a mean total functional score (TFC) of 8.6 (SD 4.5, range 0–13), and a Unified HD Rating Score (UHDRS) total motor score (TMS) of 34.6 (SD 29.6, range 0–96). Mean CSF total tau was correlated with age in healthy controls (*r* = 0.45, *p* = 0.006) and with disease burden score (a product of age and CAG repeat length) in gene expansion carriers (*r* = 0.23, *p* = 0.034). No difference was found in CSF total tau concentrations between genders (*p* = 0.436). Age‐adjusted CSF total tau was significantly elevated in gene expansion carriers compared with healthy controls (*p* = 0.002) (Fig. 1a). Total functional capacity was significantly correlated with CSF total tau (*r* = −0.29, *p* = 0.004) after adjustment for age, but not after adjustment for disease burden(*r* = −0.21, *p* = 0.146, Fig. 1b). UHDRS total motor score was significantly correlated with CSF total tau after adjustment for age (*r* = 0.32, *p* = 0.002) and also after adjustment for disease burden score (*r* = 0.30, *p* = 0.025, Fig. 1c). UHDRS symbol‐digit modality test, Stroop color matching task, and Stroop word matching task were significantly correlated with CSF total tau after adjustment for age (*n* = 24; *r* = −0.38, *p* = 0.020; *r* = −0.40, *p* = 1.335*10^−4^; *r* = −0.40, *p* = 2.426*10^−4^) and also after adjustment for disease burden score (*r* = −0.34, *p* = 0.008; *r* = 0.32, *p* = 0.002; *r* = −0.32, *p* = 0.039). Stroop interference task was not correlated with CSF total tau (*n* = 24; *r* = −0.21, *p* = 0.147). There was no association between CSF total tau and CSF hemoglobin concentration.

**Table 1:** Characteristics of included participants

Group	*N n* (%)	Age mean (SD)	Male *n* (%)	CAG mean (SD)	Burden score mean (SD)	TFC mean (SD)	TMS mean (SD)
Total	76 (100)	47.9 (13.1)	33 (43.4)	43.8 (3.9)*	389.1 (151.8)*	8.6 (4.5)*	34.6 (29.6)*
Healthy controls	24 (31.6)	45.1 (14.3)	8 (33.3)	N/A	N/A	N/A	N/A
Pre‐manifest HD	13 (17.1)	39.5 (12.5)	6 (46.2)	42.4 (2.5)	255.0 (100.5)	12.6 (0.7)	2.4 (2.7)
Early HD	22 (29.0)	49.3 (10.4)	8 (36.3)	44.1 (2.7)	402.6 (84.9)	10.8 (1.5)	28.3 (15.7)
Moderate HD	6 (7.9)	59.6 (6.2)	5 (83.3)	41.7 (2.7)	365.8 (149.3)	5.0 (0.9)	51.8 (21.1)
Advanced HD	11 (14.5)	54.5 (11.7)	6 (54.5)	46.1 (6.2)	533.4 (179.2)	1.4 (1.4)	75.7 (15.8)

HD, Huntington's disease; N/A, non‐applicable; SD, standard deviation; TFC, UHDRS total functional capacity; TMS, UHDRS total motor score *, excluding healthy controls.

## Discussion

This study confirms in two independent populations the previous finding that CSF total tau concentrations in HD gene mutation carriers are increased compared with healthy controls (Constantinescu *et al*. 2011), and the establishment of this difference persists after controlling for age – a possible confounder in the previous report.

To the best of our knowledge, ours is the first work where CSF total tau concentration has been shown to be associated with phenotypic variability in HD as measured by motor manifestations and cognitive dysfunction. These associations persist even after adjustment for disease burden score – indicating that CSF tau has independent power to predict clinical phenotype, beyond the known effects of age and CAG repeat length.

Tau is a microtubule‐associated protein, which under certain pathologic conditions, aggregates abnormally. Still, it is controversial whether these accumulations are cytotoxic and cause cellular dysfunction, contributing to neuronal cell death, or are just an epiphenomenon (Bretteville and Planel 2008). Moreover, while tau expression is particularly high in neurons with thin unmyelinated axons, this is not specific to a particular region or neuronal subpopulation and its elevation in CSF is considered a non‐specific marker of neuronal death (Zetterberg *et al*. 2013).

**Figure 2:**
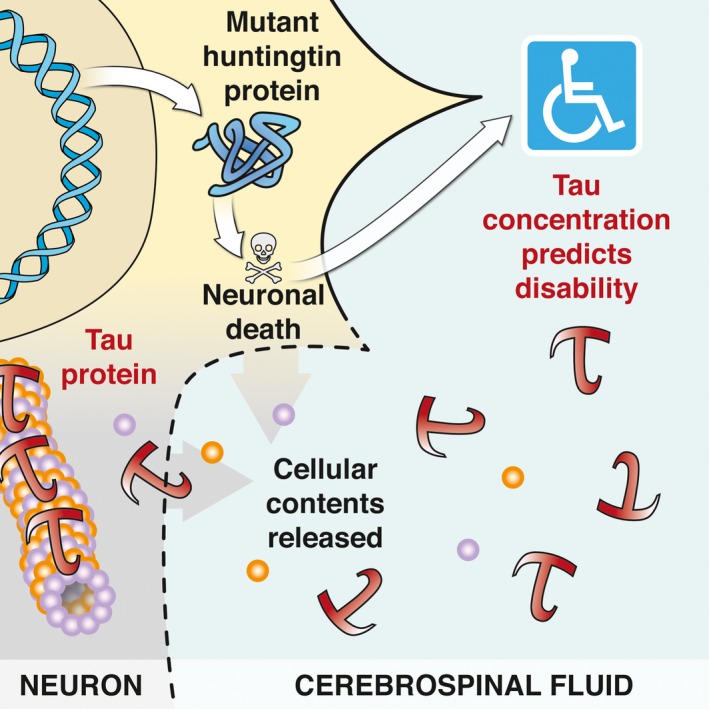
Tau is a neuronal cell death biomarker and is correlated with the disability caused by mHTT protein.

Our findings strengthen the case for CSF total tau concentration as a biomarker in HD, reflecting neuronal death and/or secondary tau pathology (Fig. 2). They suggest the need for longitudinal analysis of CSF total tau to try to establish its ability to predict disease progression, phenotypic variability and, eventually, therapeutic response. Further, this work suggests the need for further investigation of microtubule‐associated protein dynamics in HD.

## Supporting information


**Appendix S1.** Study methods.
