# Supplementary material for: Cerebrospinal fluid total tau concentration predicts clinical phenotype in Huntington's disease
Source: J Neurochem. 2016 Sep 20;139(1):22–5. doi: 10.1111/jnc.13719 (PMC5053298; doi:10.1111/jnc.13719)
Supplement: Supplementary file 1 — Appendix S1. Study methods. [file JNC-139-22-s001.docx]

**Appendix e-1**

**Study methods**

**Ethical approval:** All human experiments were performed in accordance with the declaration of Helsinki and approved by University College London (UCL)/UCL Hospitals Joint Research Ethics Committee (UK participants) and the University of British Columbia Clinical Research Ethics Board, as appropriate. All subjects gave informed written consent.

**Participants:** Subjects were recruited from two HD multidisciplinary clinics, one in London, U.K and other in Vancouver, Canada. Healthy controls, pre-symptomatic gene expansion carriers and symptomatic gene expansion carriers were included. Individual with infectious, inflammatory, or other concomitant CNS disorders or significant comorbidities were excluded. Healthy controls were defined as individuals without family history of HD and without symptoms compatible with HD, or without a CAG-expanded allele of the HD gene. Asymptomatic gene expansion carriers were defined as individuals with a CAG-expanded allele of the HD gene and with a diagnostic confidence level (DCL) inferior to 4 (Reilmann *et al.* 2014). Symptomatic gene expansion carriers were defined as individuals with a CAG-expanded allele of the HD gene and with a DCL of 4(Reilmann et al. 2014).

**Clinical assessment:** All participants underwent assessment with the Unified HD Rating Scale ’99 (UHDRS) (Huntington's disease study group 1996), assessed by an experienced neurologist. Age, gender, CAG repeat length of gene expansion carriers, disease stage (Bates *et al.* 2014), total functional capacity(Shoulson & Fahn 1979) (TFC) and total motor score (Huntington's disease study group 1996) (TMS) were recorded. The UHDRS cognitive tasks (Symbol-digit modality test, Stroop colour matching task, Stroop word matching task, and Stroop interference task) were also recorded in the Vancouver cohort (Huntington's disease study group 1996). Disease burden was calculated according to CAG repeat number and age (Penney *et al.* 1997). Patients with motor abnormalities were defined as having early, moderate, or advanced disease using the TFC scale (13-7, early; 6-4, moderate; 3-0, advanced) assessed by experienced clinical raters (Bates et al. 2014).

**CSF sample collection and storage:** CSF was obtained by lumbar puncture performed between 9:00 and 11:00 am after fasting from midnight (water was permited), examined by microscopy, and centrifuged to remove cells, and the acellular portion was frozen at -80 °C. Further details were as previously published (Wild *et al.* 2015). Haemoglobin concentration using multi-wavelength spectrophotometric readings was assessed to determine CSF contamination by blood.

**CSF total tau quantification:** CSF total tau was quantified using the INNOTEST enzyme-linked immunosorbent assay according to the manufacturer’s instructions (Fujirebio, Ghent, Belgium) in one round of experiments using one batch of reagents by board-certified laboratory technicians who were blinded to clinical data.

**Statistical analysis:** Statistical analysis was performed with Stata 14 software. Potentially confounding demographic variables (age and gender) were examined in preliminary analyses. Total tau distribution was tested for normality using skewness and kurtosis, Shapiro-Wilk and Shapiro-Francia tests. Comparisons between two groups adjusted for covariates were tested using ANCOVA. To study the association of total tau with disease progression we calculated Pearson’s and partial correlations coefficients. Bootstrapping with 1,000 repetitions was applied to non-normal variables. Significance level was defined as p<0.05.

Bibliography

Bates, G., Tabrizi, S. and Jones, L. (2014) *Huntington's disease*. Oxford University Press.

Huntington's disease study group (1996) Unified Huntington's Disease Rating Scale: reliability and consistency. Huntington Study Group. *Movement disorders : official journal of the Movement Disorder Society,* **11,** 136-142.

Penney, J. B., Jr., Vonsattel, J. P., MacDonald, M. E., Gusella, J. F. and Myers, R. H. (1997) CAG repeat number governs the development rate of pathology in Huntington's disease. *Annals of neurology,* **41,** 689-692.

Reilmann, R., Leavitt, B. R. and Ross, C. A. (2014) Diagnostic criteria for Huntington's disease based on natural history. *Movement disorders : official journal of the Movement Disorder Society,* **29,** 1335-1341.

Shoulson, I. and Fahn, S. (1979) Huntington disease: clinical care and evaluation. *Neurology,* **29,** 1-3.

Wild, E. J., Boggio, R., Langbehn, D. et al. (2015) Quantification of mutant huntingtin protein in cerebrospinal fluid from Huntington's disease patients. *The Journal of clinical investigation,* **125,** 1979-1986.
